# Effectiveness of a breastfeeding promotion intervention model based on Society ecosystems Theory for maternal women: a study protocol of randomized controlled trial

**DOI:** 10.1186/s12978-023-01719-4

**Published:** 2023-12-07

**Authors:** Yan-Qiong Ouyang, Jie Zhou, Jin-Yi Guo, Shi-Yun Wang, Xin Wang, Yi-Bei Zhou-Chen, Sharon R. Redding, Hui-Jun Chen

**Affiliations:** 1 School of Nursing, Wuhan University, 115 Donghu Road, Wuchang District, Wuhan, 430071 China; 2 Project HOPE, Washington, DC USA; 3 Department of Gynaecology and Obstetrics, Zhongnan Hospital of Wuhan University, 169 Donghu Road, Wuchang District, Wuhan, 430071 China

**Keywords:** Breastfeeding, Society ecosystems theory, Protocol, Randomized controlled trial, Nursing

## Abstract

**Background:**

Breastfeeding is recognized internationally as the most scientific and effective way to feed infants and young children. According to the World Health Organization in 2022, the exclusive breastfeeding rate within 6 months is 34.1% in China, which is still far from the goal of “more than 60% exclusive breastfeeding rate of infants within 6 months” by 2030 required by China’s State Council. It is necessary to promote breastfeeding and provide maternal breastfeeding guidance to increase exclusive breastfeeding. Factors influencing breastfeeding can be explained by the society ecosystems theory, distributed in macro, mezzo and micro systems. The interventions focused on breastfeeding promotion are mainly carried out in the health systems and services, home and family environment, community environment, work environment, policy environment or a combination of these facilities. But there is sparse research on integrating resources in the macro, mezzo and micro systems of maternal breastfeeding processes to promote breastfeeding behavior. A randomized controlled trial will test the effect of a breastfeeding promotion intervention model based on the society ecosystems theory versus usual prenatal and postnatal care on maternal and infant health and the exclusive breastfeeding rate at 6 months.

**Methods/design:**

The study is a single-blind, parallel design, randomized controlled trial with an intervention group (n = 109) and a control group (n = 109) that compares the effect of a breastfeeding promotion intervention model based on the society ecosystems theory with usual prenatal and postnatal care. The intervention covers macro- (policy, culture), mezzo- (family-hospital-community) and micro- (biological, psychological and social) systems of the maternal breastfeeding process. Infant feeding patterns, neonatal morbidity and physical and mental health of antenatal and postpartum women will be collected at baseline (28 to 35 weeks of gestation), 1-, 4-, and 6-month postpartum.

**Discussion:**

This is a multifaceted, multifactorial, and multi-environmental breastfeeding promotion strategy to help mothers and their families learn breastfeeding knowledge and skills. The study provides a new modality for adding breastfeeding interventions to prenatal and postnatal care for healthcare providers in the hospital and the community.

*Trial registration*: Chinese Clinical Trial Registry at www.chictr.org.cn, ChiCTR2300075795.

## Abstract (plain language summary)

Maternal education and support during breastfeeding can increase maternal breastfeeding self-efficacy, promote breastfeeding behaviors, and improve maternal and infant health outcomes. The interventions focused on breastfeeding promotion are mainly carried out in the health systems and services, home and family environment, community environment, work environment, policy environment or a combination of any of these facilities. But there is sparse research on integrating in multifaceted, multifactorial, and multi-environmental resources of maternal breastfeeding processes to help pregnant women and their families learn breastfeeding knowledge and skills. The current study optimizes the existing breastfeeding promotion intervention program and construct a breastfeeding promotion intervention program to correct the public’s perception of breastfeeding, increase breastfeeding self-efficacy and improve breastfeeding behavior, thus increasing the breastfeeding duration and improving maternal and infant outcomes. The program includes presenting breastfeeding-related policies and support facilities; prenatal educational sessions combined with theories and skills on breastfeeding, development of lactation, infants feeding and cares for maternal families; postnatal hands-on instruction and WeChat group peer support from hospital; home visits, group counseling and experience sharing from community and one-on-one personalized counseling throughout the intervention. The present study will be conducted to evaluate the effect of breastfeeding promotion intervention including prenatal and postnatal care on the breastfeeding duration, breastfeeding attitudes, knowledge, and self-efficacy, maternal and infant health.

## Background

Breastfeeding is recognized internationally as the most scientific and effective way to feed infants and young children. *Global strategy for infant and young child feeding*, jointly published by the World Health Organization (WHO) and the United Nations International Children’s Emergency Fund (UNICEF), clearly stated that breast milk is the most optimal food for infants and young children [1]. Infants and young children should be exclusively breastfed until 6 months of age to maintain optimal growth and development.

The exclusive breastfeeding (EBF) rate is an important indicator to measure the breastfeeding status of a country or region. According to WHO global breastfeeding data [2], the EBF rate of infants under 6 months is 40%. Only a few countries in the world have EBF rates of more than 60%. The EBF rate within 6 months in developing countries is 36% and 34.1% in China [3], with obvious regional differences. In the *Outlines for Children’s Development in China (2021–2030)*, it is clearly stated that the goal is a “more than 50% EBF rate of infants within 6 months” [4], and the *National Nutrition Plan (2017–2030)* proposed reaching 60% by 2030 [5]. In 2021, the National Health Commission of the People ‘s Republic of China and 15 other departments jointly issued the *Government action plan promoting breastfeeding(2021–2025)* [6]. According to the plan, by 2025, the awareness rate of breastfeeding core knowledge in maternal and child families should be reach more than 70%, which indicates that a low breastfeeding rate is a public health problem related to maternal and infant health, and the public’s ideological concept should be taken into account to popularize the core knowledge of breastfeeding [6]. Therefore, it is necessary to promote breastfeeding and provide maternal breastfeeding guidance to increase the EBF rate.

The interventions focused on breastfeeding promotion are mainly carried out in the health systems and services, home and family environment, community environment, work environment, policy environment or a combination of any of these facilities [7, 8]. Sinha et al. found that interventions delivered in a combination of settings by involving health systems, home and family and the community environment concurrently seemed to have higher improvements in breastfeeding rates [8]. The Baby-friendly Hospital Initiative (BFHI) is a key component of the WHO/UNICEF Global Strategy for Infant and Young Child Feeding [9], which plays a technical guidance role in the intervention [8, 10]. Adherence to the BFHI Ten Steps has a positive impact on short-term, medium-term and long-term breastfeeding outcomes [9].

China has a relatively complete medical service system. Community health centers (CHCs), such as the primary medical and health institutions in this system, can provide convenient and quick medical and health services for residents and achieve the maximum rational allocation of limited medical resources. In addition, community health service centers in China are required to provide family-based comprehensive care that integrate prevention, medical treatment, rehabilitation and health education for vulnerable groups such as women and children within the community [11]. Community support appears to be essential for sustaining breastfeeding impacts of the BFHI in the longer term [9]. This reflects that the community can play a major role in breastfeeding core knowledge and skills promotion [12].

In Chinese tradition, maternal and infant care is mainly completed by mothers or mothers-in-law in addition to husbands, and the attitudes and behaviors of husbands and other family members towards breastfeeding largely influences maternal breastfeeding behaviors, and the role of family members in breastfeeding promotion cannot be ignored [13, 14]. Breastfeeding interventions involving family members effectively increase the EBF rate and improve breastfeeding knowledge and breastfeeding attitude [15, 16].

Breastfeeding educational interventions are also an important approach to promote breastfeeding and provide assistance for women to learn about breastfeeding in advance. A systematic review and meta-analysis found that interventions provided by lecture combined with practical skills effectively reduced breast engorgement and improved the EBF rate [17]. The optimal delivery time, format and structure of the interventions have been suggested as being provided during the antenatal to postnatal period; multicomponent group education and individual breastfeeding counselling and telephone follow-up [8]; both individual and group based; and having ≥ three sessions [18]. In addition, peer counselling has been found as the most promising strategy associated with higher rates of EBF [19].

An Internet-based mobile health information platform, benefiting from its information sharing and diversity of intervention forms, provides a good channel for implementing health education and health guidance [20]. It is also conducive to carrying out personalized breastfeeding health education, improving maternal breastfeeding knowledge and skills, improving breastfeeding attitudes, reducing the rate of weaning during infancy, and increasing the rate of long-term EBF [21, 22]. But at present, Internet-based breastfeeding interventions employing e-technologies should consider improving interactions with mothers and personalizing the content of the proposed interventions [23].

**Fig. 1:**
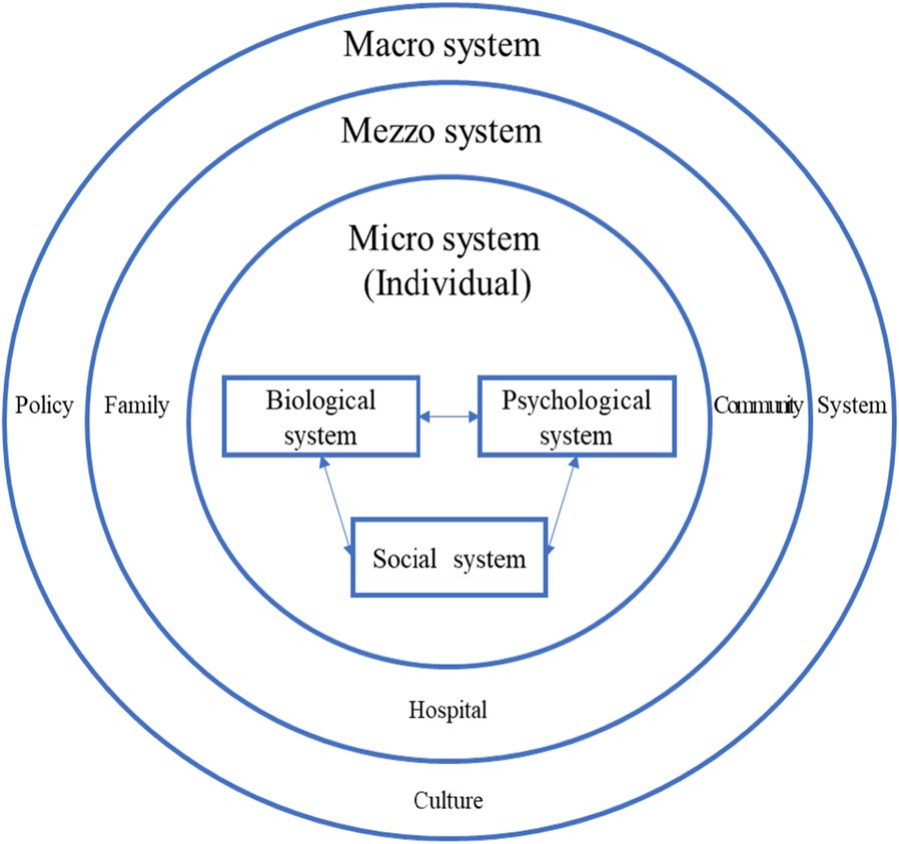
Diagram of society ecosystems theory model applied to breastfeeding

Therefore, it is necessary to construct a multifaceted, multifactorial, and multi-environmental breastfeeding promotion strategy to help pregnant women and their families learn breastfeeding knowledge and skills. According to the society ecosystems theory (SET) proposed by Zastrow [24], the author regarded the individual as a subject who develops and adapts through the interaction with various factors of the environment, and he classified the social-ecological system of the individual into three levels: micro, mezzo and macro systems. Micro system refers to an individual, which entails biological, psychological, and social systems of pregnant women in this study. Mezzo system refers to any small group, including family, work groups and other social groups. For example, hospitals, communities and families are important places for women throughout pregnancy, delivery and the postpartum period. Macro system refers to a system larger than a small group, which focuses on the social, political, and economic conditions and policies that affect people’s overall access to resources and quality of life. In the current study, this refers to the policies, institutions and culture as they relate to breastfeeding. These three systems are interrelated, mutually restrictive and influential, and all exist in the social environment. From the micro, mezzo and macro levels, efforts should be made to construct the ecological environment of breastfeeding recognition in society. The theory model applicable to breastfeeding is shown in Fig. 1.

Therefore, it is necessary to optimize the existing breastfeeding promotion intervention program and construct a breastfeeding promotion intervention program to correct the public’s perception of breastfeeding, increase breastfeeding self-efficacy and improve breastfeeding behavior, thus increasing the EBF rate and improving maternal and infant outcomes. The current study addresses to the need for an effective breastfeeding promotion intervention program that is Internet-based and that addresses the three major barriers of the micro, mezzo and macro systems regarding breastfeeding behavior.

## Methods

### Aim

This study aims to compare the effect of a breastfeeding promotion intervention model based on the SET versus a usual prenatal and postnatal care program on the EBF rate at 6 months and breastfeeding duration; on improving maternal breastfeeding attitudes, knowledge, and self-efficacy; and on improving maternal and infant health.

### Study design and setting

**Fig. 2:**
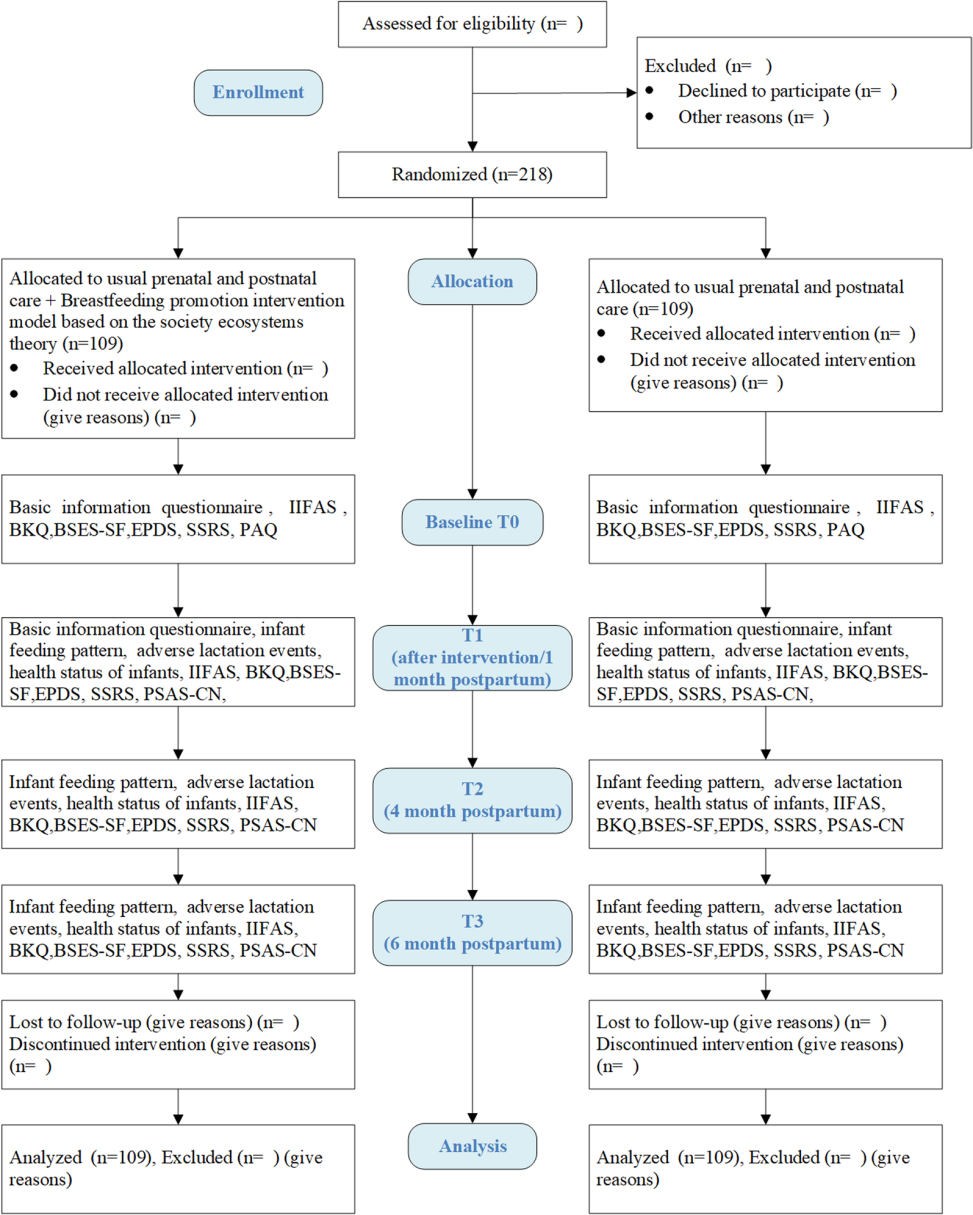
Research flow chart. *IIFAS* Iowa Infant Feeding Attitude Scale, *BKQ* Breastfeeding Knowledge Questionnaire, *BSES-SF* Breastfeeding Self-efficacy Seale Short Form, *EPDS* Edinburgh Postnatal Depression Scale, *SSRS* Social Support Rating Scale, *PAQ* Pregnancy-Related Anxiety Questionnaire, *PSAS-CN* Postpartum Specific Anxiety Scale-Chinese Version

The study is a single-blind, parallel design, randomized controlled trial with an intervention group (IG) and a control group (CG) allocated with a 1:1 randomization ratio that compares the effect of the breastfeeding promotion intervention model based on the SET, which is an IG, with a usual prenatal and postnatal care group, which is a CG (Fig. 2). This research will be conducted from August 2023 to April 2024 in Wuhan, China. Pregnant women will be recruited from August to October 2023 by convenience sampling at the Department of Obstetrics of Hubei Province Maternal and Child Health Hospital. The study protocol in compliance with the Declaration of Helsinki was approved by the Ethics Committee of Medical Research Committee of Medical College of Wuhan University (WHU-LFMD-IRB2023050) and the Hubei Province Maternal and Child Health Hospital (2023IEC070). It has registered in the Chinese Clinical Trial Registry at www.chictr.org.cn (registration number: ChiCTR2300075795).

### Eligibility criteria

Women who are aged 18 to 36 years, 28 to 35 weeks of gestation, singleton pregnancy, with basic literacy skills and no barriers to communication and able to use a smartphone will be recruited. Participation will be voluntary and informed consent will be obtained; participants can withdraw from the study at any time with no prejudice. Women with serious contraindications to breastfeeding, such as acute infectious diseases, infection with acquired immune deficiency syndrome, syphilis, mammary gland insufficiency, breast surgeries such as mastopexy; or serious mental illness are excluded.

### Sample size

The current EBF rate for infants under 6 months of age in China is 34.1% [3]. After the intervention, the rate is expected to increase to 60%, which was required by the *National Nutrition Plan (2017–2030)* [5]. Sample size was calculated for EBR in 6 months for a two-sided hypothesis test with a power of 90% and a significance level of 0.05. Therefore, according to the following formula, the study requires a minimum sample size of 76 (each group). Considering a 20% lost follow-up rate and 20% withdrawal rate because of failure to participate in the full intervention due to prematurity, a total of 218 participants will be needed for the study.

$${n}_{1}={n}_{2}=\frac{{{[u}_{\alpha }\sqrt{2P\left(1-P\right)}+{u}_{\beta }\sqrt{{P}_{1}\left({1-P}_{1}\right)+{P}_{2}\left({1-P}_{2}\right)} ]}^{2}}{{(P1-P2)}^{2}}$$

### Randomization

All patients who consent to participate and who fulfill the inclusion criteria will be randomized. Eligible participants will be randomly assigned to the IG and the CG based on a table of random numbers (https://www.sealedenvelope.com/). Results of random allocation will be placed in an opaque envelope.

### Blinding

Allocation details will be placed in opaque and sealed envelopes by a study coordinator and concealed from recruiters, data collectors and the group allocator. After recruitment and baseline data collection, the other study coordinator will open the envelopes and allocated participants. Due to the nature of the intervention, intervention conductors and participants will not be blinded to group allocation. The data collector will be masked to group allocation during data collection and the statistician will also be masked during data analyses.

### Interventions

#### Control group

Participants in the CG will receive usual prenatal and postnatal care. At the same time, they will be contacted by the researchers upon initial enrollment in the study but receive no further guidance and interventions.

#### Intervention group

The intervention of this study was constructed based on a literature review, qualitative interviews and group discussion. And the feasibility and importance of the intervention program were verified through two rounds of expert consensus based on the Delphi method [25].

Participants in the IG will be offered the breastfeeding promotion intervention model based on the SET. According to the SET, the intervention is designed in three parts: macro system, mezzo system and micro system. (1) Macro system: It mainly refers to the policies, institutions and culture that affect breastfeeding. This study can create a favorable breastfeeding atmosphere through activities such as introducing breastfeeding and childbirth-related policies and actively publicizing and reporting beneficial experiences of breastfeeding promotion. (2) Mezzo system: It refers to a small group of people including pregnant women. Hospitals, communities and families are important places for women throughout pregnancy, delivery and the postpartum period. This study will optimize the breastfeeding promotion intervention of hospital-community-family to achieve information sharing and joint management. Relying on the online WeChat mini program, it will be extended to offline services. (3) Micro system: Maternal individuals, as subjects, are the core of the social ecosystem. This study will provide social support to maternal individuals to reduce the incidence of depression and anxiety, improve the rate of breastfeeding, and promote maternal and child health. Details of the intervention are shown in Tables 1 and 2.

**Table 1:** Breastfeeding promotion intervention model based on the Society Ecosystems Theory

	Time	Content
Macro system	First week after recruitment	**Introduction to policies:** Collect breastfeeding policy documents in China and present them to pregnant women and co-parents via online images and text, such as the Global Strategy for Infant and Young Child Feeding, 10 Measures to Promote Successful Breastfeeding (2018), Action Plan for Breastfeeding Promotion (2021–2025), Breastfeeding Guidelines for Infants up to the Age of 6 Months, National Nutrition Program (2017–2030), International Code of Marketing of Breast-milk Substitutes, Chinese national and local maternity leave policies.
	First week after recruitment	** Breastfeeding support facilities:** Pregnant women and those responsible for infant care are introduced to local breastfeeding support facilities, such as mother and baby rooms, Baby Friendly Communities and Hospitals.
	Recruitment to one month postpartum	** To create a good breastfeeding atmosphere:** Actively publicize and report the beneficial experience of breastfeeding through an interactive forum in the Ai You Wei WeChat mini-program; encourage pregnant women and mothers with breastfeeding experience to join the forums and share their experiences and feelings.
Mezzo system (Family)	First week after recruitment	**Join WeChat mini-program:** Invitation to pregnant women and co-parents to join the online platform “Ai You Wei”.
	First and second week after recruitment	** Breastfeeding lecture combined with theories and skills:** Introduce the breastfeeding theory and skills course to pregnant women and co-parents through a combination of online and offline formats. Details of breastfeeding lecture are shown in Table 2.
	First and second week after recruitment	**Course evaluation:** A combination of theory and skills courses to improve breastfeeding knowledge and skills. An online questionnaire at the end of each course assesses the learning of pregnant women and co-parents and provides additional guidance.
Mezzo system (Hospital)	Recruitment to one month postpartum	** WeChat group peer support:** Establish a WeChat group jointly managed by obstetricians and gynecologists, obstetric nurses, midwives, breastfeeding counselors, and community breastfeeding professionals, and invite pregnant women and those responsible for infant care to join the group chat to obtain consultation after recruitment.
	Within 48 h after delivery	** Postnatal hands-on instruction:** Instruct mothers and infant caregivers in obstetrics wards to experience on-site breastfeeding together; insist on early contact, early suckling, and early initiation of breastfeeding; provide instruction on feeding-related skills during hospitalization, including correct feeding positions, milking techniques, and so on.
Mezzo system (Community)	Seven days, twoweeks and 1-month postpartum	** Home visits:** Researcher and community members conduct postpartum home visits to mothers to provide guidance and education to mothers and those responsible for the upbringing of infants on breastfeeding, diet, exercise, and rest for new mothers, and to improve mothers’ breastfeeding knowledge and skills.
	Delivery to one month postpartum	** Group counseling:** Provide online group counseling (four sessions) to encourage peer-to-peer communication. Mothers’ meetings are organized with the participation of obstetricians and gynecologists, obstetric nurses and researchers, and group discussions are held for mothers and co-parents, who are encouraged to speak out about their inner feelings during breastfeeding.
	Delivery to one month postpartum	**Breastfeeding experience sharing:** Mothers with breastfeeding experience are invited to share the beneficial experience of breastfeeding online every week (four sessions).
Micro system	Recruitment to one month postpartum	**Individual counseling:** One-on-one personalized education or guidance throughout the intervention through home visits, phone calls, and online communication for ongoing breastfeeding.

**Table 2:** Schedule of prenatal breastfeeding lectures including theories and skills

	Content
Theory Session 1	1. How is breast milk produced? 2. What factors affect the secretion of breast milk? 3. What are the ingredients in breast milk? 4. What are the characteristics of human milk? 5. What are the benefits of breastfeeding? 6. What do I need to prepare for breastfeeding? 7. How do I breastfeed? 8. How to tell if your baby is full?
Theory Session 2	1. How to prevent and manage common lactation problems? 2. How do mothers with flat or inverted nipples breastfeed? 3. How do mothers with sore or cracked nipples breastfeed? 4. How can breast swelling be prevented and relieved? 5. How do mothers breastfeed when they suffer from infectious/contagious diseases? 6. How should infants and young children be fed under special circumstances? 7. What should mothers eat in lactation period?
Skill Session 1	1. What are the common breastfeeding positions? 2. What are the best positions for babies to latch on to the breast and suckle? 4. What are the ways to promote milk secretion? 5. What are the ways to promote milk reflex? 6. How do I perform breast massage? 7. How do I perform hand milking?
Skill Session 2	1. how do I use a breast pump? 2. How do I store and warm breastmilk? 3. How do I breastfeed after work? 4. How do I wean properly? 5. Additional feeding knowledge of interest to pregnant mothers.

1. Format: online Tencent meeting; duration: 1 h per theory session and 40 min per skill session

2. The four sessions will be completed before the delivery, and the specific time of each class will be adjusted according to the availability of the participants and their families

3. Participants can invite at least one family member to attend each lecture

4.Lectures will be offered in small groups of five to ten participants per session

#### Intervention personnel training

Prior to the start of the study, the intervention implementers will undergo uniform training and assessment. Criteria for inclusion of intervention implementers include healthcare personnel with (1) intermediate or higher job title, (2) working in obstetrics and gynecology for ≥ 5 years, and (3) basic knowledge of breastfeeding nursing guidance. Training content and methods: (1) lectures on breastfeeding-related theories and skills; (2) self-study and online learning: theoretical knowledge and related materials, such as the *Global strategy for infant and young child feeding* [1], *Guide to infant feeding and nutrition* [26], and *Breastfeeding guidelines for infants within 6* *months of age* [27]. (3) simulation scenario: hands-on breastfeeding instruction. Assessment methods: (1) self-assessment: evaluation using the breastfeeding knowledge questionnaire (BKQ); (2) peer assessment: test questions on knowledge of breastfeeding theories and skills; (3) simulation method: simulated lectures and hands-on instruction on breastfeeding.

### Data collection

#### Basic information questionnaire

A researcher-designed questionnaire of socio-demographic information will be used to collect basic information about participants, including gestational age, number of pregnancies, parity, education level, family monthly income, employment status, length of maternity leave, co-parenting members, conception mode at baseline; and delivery mode, weeks of delivery, time of breastfeeding initiation, separation of mother and infant, and presence of comorbidities at 1 month postpartum.

#### Primary outcome

Breastfeeding rate is the primary outcome in this study. The infant feeding patterns are collected at 1-, 4- and 6-months postpartum. The breastfeeding rate and EBF rate at 1-, 4-, and 6-months postpartum are also calculated.

#### Secondary outcomes

##### Maternal and neonatal morbidity outcomes

Maternal adverse lactation events include depressed nipples, breast milk stasis, acute mastitis, cracked nipples, breast swelling, breast eczema, and nipple trauma. The health status of infants will be investigated at 1-, 4- and 6-months postpartum by asking the mothers and their co-parents whether infants suffer from any conditions (eczema, anemia, diarrhea, and pneumonia). The height and weight of the infant at birth, 1 month, 4 months, and 6 months will also be investigated by questioning the mothers and co-parents.

##### Iowa infant feeding attitude scale (IIFAS)

The IIFAS will be used to assess maternal feeding attitude. This scale includes 17 items developed by Mora and colleagues [28]. The items incorporate a five-point Likert scale with categories of “strongly disagree” (1), “disagree” (2), “being neutral” (3), “agree” (4), and “strongly agree” (5). Nine items (numbers 1, 2, 4, 6, 8, 10, 11, 14 ,17) are reverse-scored while others are positively scored. The total final score ranges from 17 (showing negative breastfeeding attitude) to 85 (indicating positive breastfeeding attitude). The original scale showed good reliability with Cronbach’s ranging from 0.68 to 0.86 and good validity [28]. In the current study, a Chinese version of the IIFAS is used and indicates moderate reliability with Cronbach’s α ranging from 0.589 to 0.685 and good validity with 0.996 [29].

##### Breastfeeding knowledge questionnaire (BKQ)

The BKQ is used to assess breastfeeding knowledge of maternal women. The questionnaire includes 18 items and was modified based on previous literature [30] by Ouyang et al. [31]. It is a closed-ended questionnaire with true or false responses and had good internal reliability with Cronbach’s α = 0.93 [31]. The common use in previous studies [31, 32] was calculating the percentages of true or false of each item. In this study, the researchers imply true as “1”and false as “0” and will then calculate the total score of the 18 items of each participant.

##### Breastfeeding self-efficacy Seale Short Form (BSES-SF)

Breastfeeding self-efficacy refers to the degree of confidence of mothers in their ability to breastfeed their infants. Breastfeeding Self-efficacy Scale (BSES) was developed by Dennis and Faux [33] in 1999 and was simplified in 2003 [34]. It was translated into Chinese by Liu et al. [35]. The Chinese version of the BSES-SF consists of 14 items with Cronbach’s α = 0.927. The scores’ range is 14 to 70 on a five-point Likert scale, with a score of 1 to 5 representing a range from “not at all” to “very confident”, with higher breastfeeding self-efficacy being associated with higher scores. The scale is divided into two dimensions: the skills dimension consists of nine items that measure the level of confidence in breastfeeding skills, and the personal internal reflection dimension of five items that measure attitudes and beliefs about breastfeeding.

##### Edinburgh postnatal depression scale (EPDS)

The EPDS is mainly used to assess the degree of postpartum depression. It was developed by Cox et al. [36], and translated into Chinese by Lee et al. [37] with a Cronbach’s α coefficient of 0.89, which showed good reliability and validity. In 2007, Guo et al. [38] translated and revised the scale according to the language habits of Chinese with a Cronbach’s α coefficient of 0.76 and the cut-off value of 9.5 [39]. It consists of 10 items, including mood, anxiety, fear, pleasure, self-blame, insomnia, coping ability, sadness, crying and self-harm. Each item has 4 points, and the total score is 30 points, with higher scores indicating more severe depression. In this study, postpartum depression was considered having a total score of ≥ 10 [40].

##### Social Support Rating Scale (SSRS)

The SSRS in a Chinses version was first developed by Xiao [41]. The scale included 10 items in three dimensions: objective support, subjective support and utilization of social support. The scores of items 1–4 and 8–10 are 1–4 points each. In item 5, the total score is scored with five options (A-E), ranging from “none “(1) to “full social support” (4). If the answer “none” in items 6 and 7 is scored for 0 points, and then the answer “other sources” is scored according to the number of sources. The scores of 35 and 45 are used as cut-off values to divide them into relatively low, general and relatively high social support. The Cronbach’s α coefficient ranged from 0.825 to 0.896, indicating good reliability of the scale [42].

##### Pregnancy-related anxiety questionnaire (PAQ)

The PAQ was developed by Chinese scholars Xiao Limin et al. [43] according to the characteristics of pregnant women in China, and is a self-assessment scale with 13 items using a 4-point scale ranging from 1 to 4. The Cronbach’s α coefficient and re-test reliability of the scale are 0.81 and 0.79, respectively. It focuses on three dimensions of worrying about the self (items 1–6), worrying about the health of the fetus (items 7–10, 13) and worrying about childbirth. The PAQ has no defined cut-off value while Zhang et al. [44] used the overall score of 75th percentile (P75) as the cut-off value for the presence or absence of pregnancy-related anxiety, i.e., 24 indicating the presence of pregnancy-related anxiety, otherwise it was none.

##### Postpartum specific anxiety scale-chinese version (PSAS-CN)

The Postpartum Specific Anxiety Scale (PSAS) was introduced in Turkey in 2016 by Assistant Professor Victoria Fallon [45]. The Chinese version was translated and revised by Xu et al. [46]. The scale consists of 50 items and contains four dimensions: (1) maternal competence and attachment anxiety; (2) infant health and safety; (3) infant care anxiety; and (4) mother’s psychosocial adjustment. The scale is a 4-point scale, with scores from 1 to 4 indicating “not at all,“ “occasionally,“ “often,“ " usually,“ and “always”. The higher the score, the more severe the symptoms of postpartum-specific anxiety. The scale has good reliability and according to Fallon et al. [45], and a score of 112 and above indicates a clinical level of anxiety. The Cronbach’s α coefficient for this scale is 0.95 [47].

#### Training and certification plans

Details of the data collection procedures are shown in Fig. 2. Collection will be completed by the same data collector, who will be trained on the study requirements, understanding of each questionnaire item, counseling for adherence and the eliciting of information from study participants in a uniform reproducible manner. The data collector will also be taught how to code each questionnaire item and how to enter data forms in the Excel spreadsheet. Each of the data collection forms and the nature of the required information will be discussed in detail on an item-by-item basis during a training session.

#### Participant withdrawal

Participants may withdraw from the study for any reason at any time. The investigator may also eliminate participants from the study in order to protect their safety and/or if they are unwilling or unable to comply with required study procedures after consultation with the researchers.

#### Data management

In the study, data will be entered electronically in an Excel file. Participant files will be stored in numerical order and stored in a secure and accessible place and manner. Participant files will be maintained in storage for a period of 3 years and uploaded to a web-based public database (ResMan research manager http://www.medresman.org.cn/pub/cn/proj/guide.aspx) upon completion of the study. The option to choose a value from a list of valid codes and a description of what each code means will be available where applicable.

### Statistical analysis

Statistical analysis will be completed using SPSS (version 24.0). In order to compare the difference at baseline between the two groups, the two-sample t-test will be used for continuous variables and the χ^2^ test for classified data. Analyses of differences between two groups regarding maternal and neonatal morbidity outcomes, IIFAS, BKQ, BSES-SF, PAQ, PSAS-CN, EPDS and SSRS are evaluated in the same manner. In addition, one-way ANOVA analysis and two-sample t-test will be used to explore the influencing factors of the EBF rate. The data from the IIFAS, BKQ, BSES-SF, PAQ, PSAS-CN, EPDS and SSRS of the two groups at different time points (T0, T1, T2 and T3) will be tested using repeated measurement Analysis of Variance (ANOVA). For variables that have an interaction effect in repeated measurement ANOVA, a simple effect analysis will be carried out to further explore the effects of each factor. Cohen’s d will be reported to check the effect size. Cohen’s d will be used to evaluate the magnitude of difference between the two groups. According to the standard established by Cohen, the large, medium and small of the effect of d was divided into 0.8, 0.5, and 0.2 [48]. The randomized trial will use the statistical analysis “per protocol”. The significance level is set to less than 0.05 (double-tailed). P-values will be reported to four decimal places p-values and those less than 0.001 will be reported as p < 0.001.

## Discussion

This randomized controlled trial will evaluate the relative effects of a SET-based breastfeeding promotion program on the EBF rate and maternal and infant health. It is hoped that the study can provide an effective approach for mothers and families to gain breastfeeding knowledge and acquire breastfeeding skills and solve breastfeeding difficulties, thus improving maternal breastfeeding attitude, knowledge and self-efficacy, increasing the rate of EBF, helping families to reduce childcare costs and promoting maternal and child health.

The theoretical advantage of this study is the usage of SET, which emphasizes the role of macro, mezzo and micro system factors and interactions between the individual and the environment in influencing individual behavior [24]. The research based on SET was conducted earlier in foreign countries. McLeroy et al. [49] applied SET to the field of health promotion and found that in order to promote the health of groups, it is necessary to consider the individual, community and social factors at the same time, which not only contains individual factors of health education activities, but also includes community social support, the development of policies, changing the environment and other aspects of the nursing strategy. The SET was widely used in the fields of obesity management [50, 51], improvement of psychological problems [52], diabetes [53] and breast cancer prevention [54], etc., which provides evidence for the application of SET to health promotion. In China, SET was mainly used in social work [55], such as mental health education of adolescents [56], school bullying [57], etc., but started late and developed slowly in the health promotion field. In 2003, Chen et al. [58] proposed to deal with health crises using ecological thinking for the first time. In recent years, Chinese researchers have applied the theory to the field of nursing, and confirmed the feasible of using SET in breast and cervical cancer [59], family management of children with leukemia [60] and diabetes [61], and caregivers of children with hearing impairment [62]. Cato et al. [63], who first applied SET to the field of breastfeeding, conducted a qualitative study based on SET to explore the attitudes of mothers-to-be towards breastfeeding, and found that women described breastfeeding as a balancing act between societal norms and their personal desires. In addition, the influence towards breastfeeding attitudes included experiences of friends and family members, acquiring knowledge about breastfeeding, healthcare professionals and perceived pressures from society towards breastfeeding [63]. Therefore, there is evidence to use SET as a theoretical model to develop interventions to address the influences of systems and promote breastfeeding behaviors.

The methodological strength of this study is that literature review and qualitative interviews were used during the intervention design of this study to synthesize the experience of previous studies and the actual needs of pregnant women to better support them. This intervention program went through two rounds of expert consensus based on the Delphi method [25], and a total of 12 experts from one university and seven hospitals completed the questionnaire based on the experts’ consensus to assess the importance and rationality of this intervention program. The strengths of the intervention are the addition of appropriate sessions based on the actual needs of the participants and the provision of individualized counseling and guidance. Blixt et al. [64] highlighted the importance of professionals providing evidence-based breastfeeding support in an individualized manner, which may enhance the positive nature of women’s breastfeeding experience.

However, this study will be conducted in only one baby-friendly hospital in a large city in central China, and a multicenter trial cannot be conducted. This might influence the generalizability of the research findings to other regions. Furthermore, due to the social desirability, women who are interested in or willing to breastfeed will participate well in this study, which may result in some biases.

Considering the large disparity in EBF rates between China and the WHO’ recommendations, such a study is needed in order to provide pregnant women with reliable breastfeeding support from the policy system, family, hospital, community and individual levels and provide a reference for countries around the world. This study synthesizes resources at all stages of a woman’s breastfeeding journey to support breastfeeding promotion and provides a new perspective for adding breastfeeding interventions to prenatal and postnatal care for hospital and community healthcare providers.

## Data Availability

Data will be uploaded to the web-based public database (ResMan research manager http://www.medresman.org.cn/pub/cn/proj/guide.aspx) after completion of the study. The datasets will be available from the corresponding author on reasonable request.

## Abbreviations

ANOVA: Analysis of variance
BFHI: Baby-friendly Hospital Initiative
BKQ: Breastfeeding Knowledge Questionnaire
BSES-SF: Breastfeeding Self-efficacy Seale Short Form
CG: Control group
CHCs: Community health centers
EBF: Exclusive breastfeeding
EPDS: Edinburgh Postnatal Depression Scale
IG: Intervention group
IIFAS: Iowa Infant Feeding Attitude Scale
PAQ: Pregnancy-Related Anxiety Questionnaire
PSAS-CN: Postpartum Specific Anxiety Scale-Chinese Version
SET: Society ecosystems theory
SSRS: Social Support Rating Scale
UNICEF: United Nations International Children’s Emergency Fund
WHO: World Health Organization
